# The relationship between the dopaminergic system and depressive symptoms in cervical dystonia

**DOI:** 10.1007/s00259-017-3664-x

**Published:** 2017-03-17

**Authors:** E. Zoons, M. A. J. Tijssen, Y. E. M. Dreissen, J. D. Speelman, M. Smit, J. Booij

**Affiliations:** 1 Department of Neurology, Academic Medical Centre, Amsterdam, The Netherlands; 2 Department of Neurology, University Medical Centre, Groningen, The Netherlands; 3 Department of Nuclear Medicine, Academic Medical Centre, PO Box 22660, 1100 DD Amsterdam, The Netherlands

**Keywords:** Cervical dystonia, SPECT, Dopamine D2/3 receptor, Dopamine transporter (DAT), Depression

## Abstract

**Purpose:**

Cervical dystonia (CD) is associated with tremor/jerks (50%) and psychiatric complaints (17–70%). The dopaminergic system has been implicated in the pathophysiology of CD in animal and imaging studies. Dopamine may be related to the motor as well as non-motor symptoms of CD. CD is associated with reduced striatal dopamine D_2/3_ (D2/3) receptor and increased dopamine transporter (DAT) binding. There are differences in the dopamine system between CD patients with and without jerks/tremor and psychiatric symptoms.

**Methods:**

Patients with CD and healthy controls underwent neurological and psychiatric examinations. Striatal DAT and D2/3 receptor binding were assessed using [^123^I]FP-CIT and [^123^I]IBZM SPECT, respectively. The ratio of specific striatal to non-specific binding (binding potential; BP_ND_) was the outcome measure.

**Results:**

Twenty-seven patients with CD and 15 matched controls were included. Nineteen percent of patients fulfilled the criteria for a depression. Striatal DAT BP_ND_ was significantly lower in depressed versus non-depressed CD patients. Higher DAT BP_ND_ correlated significantly with higher scores on the Unified Myoclonus Rating Scale (UMRS). The striatal D2/3 receptor BP_ND_ in CD patients showed a trend towards lower binding compared to controls. The D2/3 BP_ND_ was significantly lower in depressed versus non-depressed CD patients. A significant correlation between DAT and D2/3R BP_ND_ was found in both in patients and controls.

**Conclusions:**

Alterations of striatal DAT and D2/3 receptor binding in CD patients are related mainly to depression. DAT BP_ND_ correlates significantly with scores on the UMRS, suggesting a role for dopamine in the pathophysiology of tremor/jerks in CD.

## Introduction

Dystonia is characterized by sustained or intermittent muscle contractions causing abnormal, often repetitive, movements, postures, or both [1]. Idiopathic cervical dystonia (CD; dystonia of the neck) is the most common form [2]. Approximately 50% of CD patients suffer from myoclonus (jerks) or tremor of the head. It has been hypothesized that patients with tremor/jerks have a more severe phenotype, with segmental spreading of dystonia and more often an underlying genetic cause [3]. One of the regions hypothesized to be involved in the pathophysiology in tremor/jerks in dystonia is the nucleus of Cajal, which obtains information projected from the substantia nigra pars compacta, implicating the dopaminergic system [4].

Over the last few years, there has been increasing awareness of non-motor symptoms in CD patients. Psychiatric complaints, mainly depressive symptoms and anxiety disorders, have been described in a significant number of patients with dystonia (17–70%) [5–7]. Lifetime prevalence of up to 91.4% has been reported [5]. It is hypothesized that motor and psychiatric symptoms have a common underlying biochemical etiology [8, 9].

Several studies have implicated the dopaminergic system in the pathophysiology of dystonia. A hyperdopaminergic system, defined as an increased concentration of synaptic dopamine, is an attractive hypothesis in dystonia. In animal models of inherited forms such as myoclonus dystonia (M-D) and DYT1 dystonia, a hyperdopaminergic system has been confirmed [10, 11].

Human studies have shown lower striatal dopamine D_2/3_ (D2/3) receptor binding in patients with CD, writer’s cramp and M-D [12–15]. According to the competition model, this decreased D2/3 receptor binding is compatible with higher concentrations of synaptic dopamine and occupancy of more postsynaptic D2/3 receptors, a reduced number of these receptors, or a combination of both. Increased levels of synaptic dopamine may lead to upregulation of the dopamine transporter (DAT) to ensure greater reuptake of endogenous dopamine. However, previous imaging studies investigating DAT binding found no differences between dystonia patients and controls [8, 15]. Recent animal studies have shown that the dopaminergic tone is probably regulated by the amount of DAT present at the presynaptic cell membrane [16, 17]. This could explain the lack of DAT binding abnormalities found despite indications of a hyperdopaminergic system, i.e. more DAT are present, but they are occupied by the higher level of intrasynaptic dopamine.

The dopaminergic system is also implicated in psychiatric conditions, especially in major depression. Two positron emission tomography (PET) studies found reduced striatal DAT binding in patients with major depression [18, 19]. Striatal DAT binding is also negatively related to depressive symptoms in patients with Parkinson’s disease [20]. Previous nuclear imaging studies in dystonia did not correct for psychiatric symptoms.

In the present study, to further establish the role of dopamine in dystonia and comorbid psychiatric symptoms, we imaged both the presynaptic striatal DAT and the postsynaptic striatal D2/3 receptors in the same sample. We hypothesized that CD is associated with reduced striatal D2/3 receptors and increased striatal DAT binding. In addition, we investigated whether there were differences in the dopamine system between patients with and without jerks/tremor and between patients with and without psychiatric symptoms.

## Material and methods

### Subjects

We included patients who had been previously diagnosed with idiopathic CD by an experienced neurologist. Neurological examination and additional tests (laboratory tests, genetic tests and conventional imaging) revealed no signs of acquired or inherited dystonia (including dopa-responsive dystonia). Inclusion criteria were as follows: CD that had been stable according to the Tsui scale for at least 1 year during botulinum neurotoxin (BoNT) treatment [21], and age of 35–80 years. BoNT injections were administered on the day of single-photon emission computed tomography (SPECT) scanning or a maximum of 7 days prior to/after scanning. This applied for both scans. Scans were acquired within 3 to 7 days of each other. Exclusion criteria were other relevant neurological conditions at inclusion or in the past, treatment with deep brain stimulation (DBS), use of antidepressants in the past 6 months, symptomatic therapy for dystonia other than BoNT and low dosages of benzodiazepines, use of medication with a known dopaminergic or serotonergic effect [22], and pregnancy or lactation. Patients were allowed to use other medications, e.g. antihypertensive drugs. Healthy age- and sex-matched subjects (recruited through flyers in the hospital) served as the control group. Controls had a normal neurological examination and no self-reported history or family history of dystonia, myoclonus or psychiatric illness. Written informed consent was obtained from all subjects, and the study was approved by the local medical ethics committee.

### Scoring neurological and psychiatric symptoms

The neurological examination of patients was videotaped and blindly scored by two independent clinicians. Dystonic symptoms were scored using the Toronto Western Spasmodic Torticollis Rating Scale (TWSTRS) [23] and the Tsui scale [21]. Symptoms of myoclonus were scored using the Unified Myoclonus Rating Scale (UMRS) [24]. The independent scores on the Tsui and TWSTRS revealed good agreement between the two observers (>0.80 intraclass correlation coefficients, two-way mixed, absolute agreement, average measures). The independent scores on the UMRS had an intraclass correlation coefficient of 0.73. The average scores of the two experts on the Tsui, TWSTRS and UMRS were used in the statistical analysis. Subjects completed several take-home questionnaires concerning psychiatric symptoms. The psychiatric interview, performed by a trained investigator (EZ, YD), consisted of the Mini International Neuropsychiatric Interview (MINI)-Plus and several questionnaires concerning symptoms of depression and anxiety. For this study we incorporated the results of the Beck Depression Inventory (BDI; home questionnaire) and Montgomery–Asberg Depression Rating Scale (MADRS; incorporated in psychiatric interview) for depression and the Liebowitz Social Anxiety Scale (LSAS; home questionnaire) and the Beck Anxiety Inventory (BAI; home questionnaire) for anxiety. Subjects were judged to have a depressive disorder when they fulfilled the relevant criteria on the MINI and/or had a MADRS score ≥ 20 points or BDI score ≥ 14 points. These cut-off values correspond to moderate–severe depression. Subjects were judged to have an anxiety disorder when they fulfilled the relevant criteria on the MINI-Plus and/or had a BAI score ≥ 16 points or LSAS score ≥ 30 points. These cut-off values correspond to moderate–severe anxiety.

### SPECT imaging

All participants received 300 mg potassium iodide to block thyroid uptake of free radioactive iodide before administration of the tracer. For the DAT study, subjects received a mean dose of 100 MBq of [^123^I]FP-CIT intravenously (produced according to good manufacturing practices [GMP] criteria by GE Healthcare) as a bolus [25]. Scans were performed 3 h after bolus injection to visualize and quantify the specific DAT binding in the striatum [26]. For visualizing striatal D2/3 receptor binding, subjects received a 56 MBq bolus of [^123^I]IBZM intravenously (produced according to GMP criteria by GE Healthcare) followed by continuous infusion of 14 MBq/h of [^123^I]IBZM until the end of the scan to achieve unchanging regional brain activity levels [27, 28]. Acquisition of the images was started 2 h after the bolus injection [27, 29]. SPECT studies were performed using a 12-detector single-slice brain-dedicated scanner (Neurofocus 810 is an upgrade for the Strichman Medical Equipment 810X camera) with a full-width at half-maximum resolution of approximately 6.5 mm throughout the 20-cm field of view. After positioning of the subjects with the head parallel to the orbitomeatal line, axial slices parallel and upward from the orbitomeatal line to the vertex were acquired in 5-mm increments, with an average of 15 slices in a 64×64 matrix. Scanning time was 3.5 min per slice for [^123^I]FP-CIT and 5 min per slice for [^123^I]IBZM SPECT. The energy window was set at 140–178 keV. Images were reconstructed in 3-D mode and analysed blindly by one observer (EZ). For the [^123^I]FP-CIT SPECT images, fixed regions of interest (ROIs) for caudate nucleus and putamen were positioned on the four consecutive axial slices with highest striatal activity, as described previously [30]. The activity in the separate ROIs was combined to reflect average activity in the caudate nucleus (left + right), putamen (left + right) and whole striatum bilaterally. The cerebellum was used as reference region by positioning an ROI, as described previously [31]. For the [^123^I]IBZM images, fixed ROIs were positioned for the striatum, as described previously [32]. The four slices with the highest striatal activity were pooled, and the average activity was calculated. An ROI was positioned on the occipital cortex on the same four slices as reference region for the IBZM tracer. For both scans, ratios of specific to non-specific binding were calculated as [(activity in ROI − activity in reference region)/activity in reference region], representing the binding potential (BP_ND_) [33]. BP_ND_ is a combined measure of the density of available neuroreceptors and tracer affinity to the neuroreceptor.

### Statistical analysis

The Mann–Whitney *U* test and Kruskal–Wallis test were used to assess differences in receptor/transporter binding ratios (BP_ND_) between different groups of subjects. The Kruskal–Wallis test was also used to assess differences in baseline characteristics between patients with and without jerks/tremor and controls. Chi-square and Fisher’s exact tests were used to assess dichotomous variables.

Linear regression was used to determine whether differences in baseline characteristics explained differences in BP_ND_, both between patients with and without jerks/tremor and between dystonia patients and healthy controls. Linear regression was also used for assessing relationships between BP_ND_ and motor and psychiatric scores and between motor and psychiatric scores. Multicollinearity among variables was avoided by categorizing motor and psychiatric symptoms and not using more than one such variable in the model. Analyses were carried out using SPSS version 20 software (IBM Corp., Armonk, NY, USA), and differences were considered significant at *p* < 0.05.

## Results

### Clinical characteristics

We included 27 patients with CD (15 with jerks/tremor and 12 without) and compared them to 15 age- and gender-matched healthy controls. Due to technical difficulties, one [^123^I]FP-CIT SPECT scan of a control and three [^123^I]FP-CIT scans and one [^123^I]IBZM scan of patients had to be excluded from the analysis. Baseline characteristics are depicted in Table 1 for subjects in whom at least one scan was available for analysis. Patients with jerks/tremor were slightly but not significantly younger than patients without jerks/tremor and controls. Tsui scores were slightly higher in patients with jerks/tremor. UMRS scores were significantly higher in patients with tremor/jerks, but even ten patients classified as having no tremor/jerks occasionally exhibited myoclonus, with UMRS scores around 1–2. Psychiatric comorbidity was common in CD patients (17/27 patients; 63%). There was no significant difference in psychiatric comorbidity between patients with and without tremor/jerks. There was no correlation between motor scores and psychiatric comorbidity, excluding multicollinearity in further regression models. Two out of 14 controls (14%) fulfilled the criteria for a psychiatric diagnosis (one for alcohol abuse in the past, and one scored 34 on the LSAS, meeting the criteria for social anxiety disorder).

**Table 1:** Baseline characteristics

Characteristics	CD with jerks/tremor (*n* = 15)	CD without jerks/tremor (*n* = 12)	Controls (*n* = 15)	*p* value
Age, years, median (IQR)	62 (53–67)	52.5 (44.5–60)	61 (56–62)	0.06
Men, *n* (%)	7 (47%)	5 (42%)	7 (47%)	0.96
Tsui, median (IQR)	8 (6.5–12)	7.8 (5.4–13.5)	N/A	0.79
TWSTRS total, median (IQR)	16.5 (14.5–21)	14.5 (13.5–20.5)	N/A	0.59
UMRS, median (IQR)	12.5 (7–19)	1.3 (0.5–2.4)	N/A	<0.001
Psychiatric disorders, *n* (%)	9 (60%)	8 (67%)	2 (13%)	<0.01
Anxiety disorders, *n* (%)	7 (47%)	6 (50%)	1 (7%)	0.02
Depression, *n* (%)	2 (13%)	3 (25%)	0 (0%)	0.13
BDI, median (IQR)	5 (2–8)	4.5 (2.25–8.5)	2 (0–3)	0.02
MADRS, median (IQR)	2 (0–4)	3.5 (0–8.5)	1 (0–2)	0.06
LSAS, median (IQR)	11 (5–34)	16 (5.5–40.25)	4 (1–8)	0.02
BAI, median (IQR)	6 (4–10)	4 (0.25–11)	1 (0–1)	<0.01

*BAI* Beck Anxiety Inventory, *BDI* Beck Depression Inventory, *CD* cervical dystonia, *IQR* interquartile range, *LSAS* Liebowitz Social Anxiety Scale, *MADRS* Montgomery–Asberg Depression Rating Scale, *n* number, *TWSTRS* Toronto Western Spasmodic Torticollis Rating Scale, *UMRS* Unified Myoclonus Rating Scale

### [^123^I]FP-CIT SPECT – dopamine transporter imaging

There was no difference between DAT BP_ND_ in the whole striatum between CD patients (3.48; IQR 3.01–3.84) and controls (3.64; IQR 3.33–3.99; *p* = 0.41) or in the caudate nucleus (p = 0.58) or putamen (*p* = 0.38) separately. DAT BP_ND_ was comparable between patients with and without jerks/tremor for the whole striatum (*p* = 0.24), caudate nucleus (*p* = 0.33) and putamen (*p* = 0.37). Neither Tsui (*p* = 0.86) nor TWSTRS (*p* = 0.56) explained the variance in DAT BP_ND_. However, UMRS scores contributed significantly to differences in DAT BP_ND_ (*p* = 0.04, *r*_s_ = 0.19).

CD patients with comorbid depression had lower DAT BP_ND_ (3.02; IQR 2.51–3.39) in the whole striatum compared to patients without depression (3.54; IQR 3.34–4.03; *p* = 0.05). This difference was also present in the caudate nucleus (DAT BP_ND_ 3.00 [IQR 2.58–3.51] vs. 3.84 [IQR 3.51–4.24]; p = 0.02). DAT BP_ND_ was also lower in the putamen of patients with a depression compared to patients without, but this did not reach statistical significance (*p* = 0.14). There was no significant difference in DAT BP_ND_ between CD patients without comorbid depression and controls (DAT BP_ND_ 3.54 [3.34–4.03] vs. 3.64 [IQR 3.33–3.99]; *p* = 0.86 for whole striatum). No differences were found between patients with and without psychiatric co-morbidity and with and without an anxiety disorder. Scores of CD patients on the BDI (*p* = 0.90), MADRS (0.29), LSAS (0.81) and BAI (0.44) did not contribute to differences in DAT BP_ND_ in the whole striatum.

Since age and sex are known to have an effect on DAT BP_ND_ as measured with [^123^I]FP-CIT SPECT [34–36], and age differed slightly between groups, they could be potential confounders. Therefore, we corrected for these factors for the striatum as a whole, which did not change any of the results (regression coefficients and *p* values are depicted in Table 2). Correcting for the occurrence of depression changed the correlation between DAT BP_ND_ and UMRS scores only slightly (*p* = 0.04 before correction and 0.06 after correction).

**Table 2:** Regression analyses for DAT BP_ND_ corrected for age and sex

	Regression coefficient for DAT BP_ND_ before correction	*p* value before correction	Regression coefficient for DAT BP_ND_ corrected for age and sex	*p* value corrected for age and sex
Patients vs controls (95% CI)	0.14 (−0.71 to 0.99)	0.74	0.23 (−0.66 to 1.11)	0.61
Jerks vs no jerks (95% CI)	0.75 (−0.58 to 2.08)	0.25	0.63 (−0.84 to 2.10)	0.38
Psychiatry vs no psychiatry (95% CI)	0.33 (−1.06 to 1.72)	0.62	0.37 (−1.10 to 1.84)	0.60
Anxiety vs no anxiety (95% CI)	0.88 (−0.45 to 2.22)	0.18	0.93 (−0.47 to 2.33)	0.18
Depression vs no depression (95% CI)	−0.96 (−2.54 to 0.62)	0.22	−1.13 (−2.82 to 0.56)	0.18

### [^123^I]IBZM SPECT - dopamine D_2/3_ receptor imaging

There was a trend towards lower striatal D2/3 receptor BP_ND_ in patients with CD (0.84; IQR 0.63–0.99) compared to controls (0.91; 0.79–1.12; *p* = 0.14). There was no difference in D2/3 receptor BP_ND_ between patients with and without jerks (*p* = 0.54). Scores on the Tsui (*p* = 0.92), TWSTRS (*p* = 0.74) and UMRS (*p* = 0.67) did not contribute to differences in D2/3 receptor BP_ND_.

Patients with psychiatric symptoms did not differ in D2/3 receptor BP_ND_ from patients without (*p* = 0.20), nor did patients with an anxiety disorder differ from patients without (*p* = 0.72). Patients with depression had a significantly lower D2/3 receptor BP_ND_ (0.56, IQR 0.48–0.72) compared to patients without (0.89; IQR 0.70–1.01; *p* = 0.008). There was no significant difference in D2/3 receptor BP_ND_ between CD patients without comorbid depression and controls (D2/3 receptor BP_ND_ 0.89 [IQR 0.70–1.00] vs. 0.91 [0.79–1.12]; *p* = 0.43). In CD patients, scores on BDI (*p* = 0.25), MADRS (0.82), LSAS (0.36) and BAI (0.70) did not contribute to D2/3 receptor BP_ND._

Since age and sex are known to have an effect on D2/3 receptor BP_ND_ [34], we corrected for these factors. This did not change the result between patients and controls (*p* = 0.09 before and after correction) or between patients with and without jerks (*p* = 0.66 before correction and 0.44 after correction).

Because of the surprising finding of a large difference in D2/3 receptor BP_ND_ between patients with and without comorbid depression, we also separately corrected the D2/3 receptor BP_ND_ for the occurrence of depression. Before correction, there was a regression coefficient of −0.12 between patients and controls (95% CI −0.25 to 0.02; *p* = 0.09), and after correction the regression coefficient was −0.06 (95% CI −0.19 to 0.07; *p* = 0.34).

### DAT-D2/3 receptor ratio

**Fig. 1:**
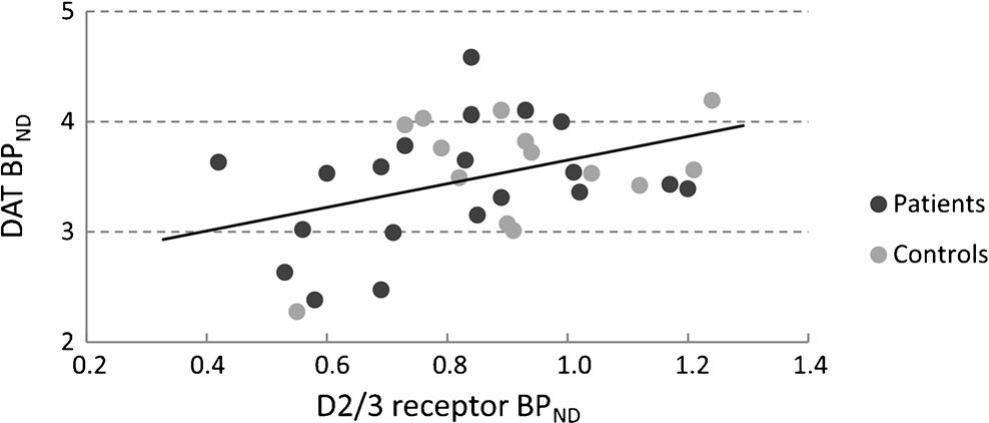
The correlation between DAT and D2/3 receptor BP_ND_ in both patients (*black circles*) and controls (*grey circles*). The D2/3 receptor BP_ND_ is shown on the *x*-axis, and the DAT BP is shown on the *y*-axis. Every black or grey circle is an individual study subject. Values are depicted only for subjects in which both a DAT and D2/3 receptor scan was performed

In patients with a lower striatal DAT BP_ND_, there was a trend towards a lower striatal D2/3 receptor BP_ND_ (regression coefficient 0.93 [95% CI −0.30 to 2.14]; *p* = 0.13). The same trend was found in controls (regression coefficient 1.03 [95% CI −0.55 to 2.61]; *p* = 0.18). When patients and controls were combined, this resulted in a statistically significant correlation between striatal DAT and D2/3 receptor BP_ND_ (regression coefficient 0.98 [95% CI 0.12–1.84]; *p* = 0.03; Fig. 1). This correlation was not caused by an age effect. When plotting the binding potential and age in graphs, in both cases the fit lines are almost horizontal but with a trend towards decreasing BP_ND_ at higher age (*R* ^2^ 0.028 for D2/3 receptor and *R* ^2^ 0.015 for DAT).

## Discussion

This study showed a strong relation between depressive symptoms and alterations in striatal DAT and D2/3 receptor binding in patients with CD. In addition, the association between DAT BP_ND_ and scores on the UMRS suggests a role for dopamine in the pathophysiology of tremor/jerks in CD.

In line with previous studies, no difference in striatal DAT binding was detected between patients and controls. Differences in DAT binding are likely to be small, but can still be clinically significant. The fact that we did not find a difference in striatal DAT binding may be explained by various factors. First, it might mean that there is no difference in the number of DATs. Second, there could be a hyperdopaminergic system that cannot be detected with DAT imaging. Two recent animal studies hypothesized that dopaminergic tone is regulated by the amount of DAT present at the presynaptic cell membrane. More intrasynaptic dopamine would lead to more DATs to bind dopamine. In that case, the amount of DAT free to bind [^123^I]FP-CIT might be stable, and no difference would be found in DAT BP_ND_ [16, 17]. Lastly, it could mean that there is a difference in intrasynaptic dopamine, but [^123^I]FP-CIT is less sensitive in detecting differences in dopaminergic concentrations compared to [^123^I]IBZM.

We did find a strong trend towards reduced striatal D2/3 receptor binding in patients with idiopathic CD compared to controls. This could be consistent with a hyperdopaminergic state in the striatum and/or with a reduced number of D2/3 receptors. Previous reports have been ambiguous about D2/3 receptor binding in dystonia, although reduced binding has been more commonly reported [13, 15, 37].

The existence of depressive symptoms within the group of CD patients was associated with a significant difference in striatal D2/3 receptor and DAT BP_ND_. There was no significant difference in DAT or D2/3 receptor BP_ND_ between non-depressed CD patients and controls. Moreover, when we corrected for comorbid depression, there was no longer a difference in D2/3 receptor BP_ND_ between patients and controls, indicating that changes in the dopamine system of CD patients may correlate mainly with depressive symptoms and not with dystonia per se. Psychiatric symptoms, more specifically anxiety and depression, are common in dystonia. In our cohort, 63% of patients had psychiatric symptoms. This is on the high end of the 17–70% range reported in observational cohort studies in the literature [6, 7, 38, 39]. Molecular imaging studies in depression and anxiety disorders have shown abnormalities in both striatal DAT and D2/3 receptor binding. Most studies in major depressive disorder (MDD) have reported decreased striatal DAT binding compared to controls [40–42]. Reduced DAT binding has also been described in anxiety disorders, although less consistently [43, 44]. Results on striatal D2/3 receptor binding in patients with depression and anxiety have been ambiguous. Abnormalities have been found, but differences with controls were smaller than observed in studies on DAT binding, and both decreased and increased binding have been described [45–47]. Reduced striatal DAT binding has been found in Parkinson’s patients with depression compared to non-depressed Parkinson’s patients, and binding in the caudate nucleus was negatively related to the severity of depressive symptoms in patients with Parkinson’s disease [20, 48]. We also observed that decreased DAT binding in the caudate nucleus was related to depressive symptoms in CD.

Another recent study investigated striatal DAT availability in different groups of patients with movement disorders and found normal DAT binding in dystonia patients, with an inverse correlation between DAT availability in the left putamen and severity of both anxiety and depression [8]. The authors hypothesized that dysfunction of the basal ganglia-thalamocortical circuits underlies both motor and psychiatric manifestations in movement disorders [8]. We hypothesize that the differences we found in striatal DAT and D2/3R binding are mainly driven by the psychiatric symptoms in dystonia. However, an effect of motor symptoms cannot be completely ruled out, especially since a recent study found differences in spatial reorganization of putaminal D2/3 receptor binding between patients with blepharospasm and hand dystonia [49]. We were unable to measure spatial redistribution due to the limited spatial resolution of SPECT imaging. We can say, however, that psychiatric symptoms play an important role in abnormalities in the dopamine system of patients with CD and should be taken into account in future imaging studies. It could be that patients who suffer from both motor and psychiatric symptoms have a more severe phenotype in which dopamine plays a more important role.

There was no difference in D2/3 receptor or DAT binding between CD patients with and without tremor or jerks, although DAT binding did correlate with scores on the UMRS [24]. This is probably because most patients classified as having no jerks (10/12) did have some mild jerks or tremor. As stated above, the nucleus of Cajal, which receives input from the substantia nigra pars compacta, has been hypothesized to play a role in the occurrence of tremor and jerks in dystonia. Biochemical changes in this region may lead to changes in DAT binding and tremor or jerks in CD patients [4]. However, this area is too small to assess in vivo in humans.

The other interesting finding is the positive correlation between DAT and D2/3 receptor binding. This is not consistent with the competition model. It is likely that the competition model applies only to acute interventions and not to chronic disease conditions such as dystonia. The relation between DAT and D2/3 receptor binding has not been studied extensively in healthy controls or in different conditions, although a recent study showed a significant positive correlation between striatal DAT and D2/3 receptor binding in healthy controls [34], using the same radiotracers we used.

This study has several limitations. The use of dopaminergic or serotonergic medication [22] was an exclusion criterion in our study, which could have excluded patients with severe psychiatric complaints, leading to an underestimation of the effect of psychiatric symptoms on DAT and D2/3 receptor binding. Patients in our study did receive BoNT injections and were allowed to use low dosages of benzodiazepines. Furthermore, since subjects were on average 50–60 years of age, most of them used medication for other conditions. BoNT is a locally acting neurotoxin without systemic effects, and its effect is noticeable after 1 week [50]. Both scans were performed before this effect could occur; thus it is unlikely that BoNT had a direct effect on striatal DAT or D2/3 binding ratios. We cannot rule out a placebo effect of the BoNT injections, and such an effect has not been investigated to date. There are some indications that benzodiazepines have an effect on D2/3 receptor binding in the striatum and dorsolateral prefrontal cortex. However, this has been investigated only with lorazepam doses sufficient to cause sedation [51, 52]. Patients in our study used a low dosage of oxazepam or clonazepam. Therefore, we do not believe that this influenced our results significantly. [^123^I]FP-CIT is derived from cocaine and metabolized by cytochrome P450 type 3A (CYP3A) in the liver [22]. The same enzyme also metabolizes most drugs. Therefore, at least theoretically, many drugs might influence [^123^I]FP-CIT metabolism and possibly striatal DAT binding. For most drugs, potential effects have not been investigated. The only potential influence we found was codeine, which was used by one of our patients in a combination drug with acetaminophen to treat pain (acetaminophen 500 mg + codeine 10 mg three times daily). Opioid abuse, including abuse of codeine, has been associated with lower DAT binding in the striatum. In one study, DAT binding correlated with the amount of opioids used [53–56]. It is unlikely that a low dosage of codeine in one patient influenced our results. Even less is known about interactions between drugs and [^123^I]IBZM binding.

Another potential weakness of this study is the fact that we did not correct for other factors that might influence the dopamine system, e.g. smoking, season and amount of sunlight exposure [57, 58]. All of these factors have been hypothesized to influence the dopamine system, although the relationship is still under debate, for example, with smoking [59]. Our study group was too small to correct for every factor that could potentially influence the dopamine system. Also, with the technique we used, it is only possible to adequately measure DAT and D2/3 receptors in the striatum. Therefore, we cannot exclude dopaminergic changes elsewhere in the brain. Lastly, the significant number of scans that had to be excluded from the analysis is a limitation. We had some technical difficulties during the course of this study, leading to poor scan quality. Even taking this into account, ours is still the largest SPECT imaging study in patients with dystonia thus far, and was the first to find that depressive symptoms likely explain differences in striatal DAT and D2/3 receptor BP_ND_ between CD patients and controls.

## References

[1] Albanese A (2013). Phenomenology and classification of dystonia: a consensus update. Mov Disord.

[2] Tarsy D, Simon DK (2006). Dystonia. N Engl J Med.

[3] Rubio-Agusti I (2013). Tremulous cervical dystonia is likely to be familial: clinical characteristics of a large cohort. Parkinsonism Relat Disord.

[4] Shaikh AG, Zee DS, Jinnah HA (2015). Oscillatory head movements in cervical dystonia: dystonia, tremor, or both?. Mov Disord.

[5] Gundel H (2003). High psychiatric comorbidity in spasmodic torticollis: a controlled study. J Nerv Ment Dis.

[6] Hess CW (2007). Myoclonus-dystonia, obsessive-compulsive disorder, and alcohol dependence in SGCE mutation carriers. Neurology.

[7] Smit M (2016). Psychiatric co-morbidity is highly prevalent in idiopathic cervical dystonia and significantly influences health-related quality of life: results of a controlled study. Parkinsonism Relat Disord.

[8] Di Giuda D (2012). Dopaminergic dysfunction and psychiatric symptoms in movement disorders: a 123I-FP-CIT SPECT study. Eur J Nucl Med Mol Imaging.

[9] Lehn A, Mellick G, Boyle R (2014). Psychiatric disorders in idiopathic-isolated focal dystonia. J Neurol.

[10] Yokoi F (2006). Myoclonus, motor deficits, alterations in emotional responses and monoamine metabolism in epsilon-sarcoglycan deficient mice. J Biochem.

[11] Song CH (2012). Functional analysis of dopaminergic systems in a DYT1 knock-in mouse model of dystonia. Neurobiol Dis.

[12] Berger HJ (2007). Writer’s cramp: restoration of striatal D2-binding after successful biofeedback-based sensorimotor training. Parkinsonism Relat Disord.

[13] Beukers RJ (2009). Reduced striatal D2 receptor binding in myoclonus-dystonia. Eur J Nucl Med Mol Imaging.

[14] Horstink CA (1997). Low striatal D2 receptor binding as assessed by [123I]IBZM SPECT in patients with writer’s cramp. J Neurol Neurosurg Psychiatry.

[15] Naumann M (1998). Imaging the pre- and postsynaptic side of striatal dopaminergic synapses in idiopathic cervical dystonia: a SPECT study using [123I] epidepride and [123I] beta-CIT. Mov Disord.

[16] Ferris MJ (2012). Cocaine self-administration produces pharmacodynamic tolerance: differential effects on the potency of dopamine transporter blockers, releasers, and methylphenidate. Neuropsychopharmacology.

[17] Lohr KM (2014). Increased vesicular monoamine transporter enhances dopamine release and opposes Parkinson disease-related neurodegeneration in vivo. Proc Natl Acad Sci U S A.

[18] Anand A (2011). Striatal dopamine transporter availability in unmedicated bipolar disorder. Bipolar Disord.

[19] Meyer JH (2001). Lower dopamine transporter binding potential in striatum during depression. Neuroreport.

[20] Vriend C (2014). Reduced dopamine transporter binding predates impulse control disorders in Parkinson’s disease. Mov Disord.

[21] Tsui JK (1986). Double-blind study of botulinum toxin in spasmodic torticollis. Lancet.

[22] Booij J, Kemp P (2008). Dopamine transporter imaging with [(123)I]FP-CIT SPECT: potential effects of drugs. Eur J Nucl Med Mol Imaging.

[23] Tarsy D (1997). Comparison of clinical rating scales in treatment of cervical dystonia with botulinum toxin. Mov Disord.

[24] Frucht SJ (2002). The unified myoclonus rating scale. Adv Neurol.

[25] Booij J (1998). Imaging of dopamine transporters with iodine-123-FP-CIT SPECT in healthy controls and patients with Parkinson’s disease. J Nucl Med.

[26] Booij J, Knol RJ (2007). SPECT imaging of the dopaminergic system in (premotor) Parkinson’s disease. Parkinsonism Relat Disord.

[27] Booij J (1997). Assessment of endogenous dopamine release by methylphenidate challenge using iodine-123 iodobenzamide single-photon emission tomography. Eur J Nucl Med.

[28] Laruelle M (1995). SPECT imaging of striatal dopamine release after amphetamine challenge. J Nucl Med.

[29] Boot E (2008). AMPT-induced monoamine depletion in humans: evaluation of two alternative [123I]IBZM SPECT procedures. Eur J Nucl Med Mol Imaging.

[30] Booij J (1997). [123I]FP-CIT SPECT shows a pronounced decline of striatal dopamine transporter labelling in early and advanced Parkinson’s disease. J Neurol Neurosurg Psychiatry.

[31] Koopman KE (2012). Assessing the optimal time point for the measurement of extrastriatal serotonin transporter binding with 123I-FP-CIT SPECT in healthy, male subjects. J Nucl Med.

[32] Figee M (2014). Deep brain stimulation induces striatal dopamine release in obsessive-compulsive disorder. Biol Psychiatry.

[33] Innis RB (2007). Consensus nomenclature for in vivo imaging of reversibly binding radioligands. J Cereb Blood Flow Metab.

[34] Jakobson Mo S (2013). (1)(2)(3)I-FP-Cit and 123I-IBZM SPECT uptake in a prospective normal material analysed with two different semiquantitative image evaluation tools. Nucl Med Commun.

[35] Lavalaye J (2000). Effect of age and gender on dopamine transporter imaging with [123I]FP-CIT SPET in healthy volunteers. Eur J Nucl Med.

[36] Varrone A (2013). European multicentre database of healthy controls for [123I]FP-CIT SPECT (ENC-DAT): age-related effects, gender differences and evaluation of different methods of analysis. Eur J Nucl Med Mol Imaging.

[37] Hierholzer J (1994). Dopamine D2 receptor imaging with iodine-123-iodobenzamide SPECT in idiopathic rotational torticollis. J Nucl Med.

[38] Gundel H (2007). Psychiatric comorbidity in patients with spasmodic dysphonia: a controlled study. J Neurol Neurosurg Psychiatry.

[39] Lencer R (2009). Primary focal dystonia: evidence for distinct neuropsychiatric and personality profiles. J Neurol Neurosurg Psychiatry.

[40] Camardese G (2014). Changes of dopamine transporter availability in depressed patients with and without anhedonia: a 123I-N-omega-fluoropropyl-carbomethoxy-3beta- (4-Iodophenyl)tropane SPECT study. Neuropsychobiology.

[41] Savitz JB, Drevets WC (2013). Neuroreceptor imaging in depression. Neurobiol Dis.

[42] Wu CK (2013). No changes in striatal dopamine transporter in antidepressant-treated patients with major depression. Int Clin Psychopharmacol.

[43] Furmark T (2009). Neurobiological aspects of social anxiety disorder. Isr J Psychiatry Relat Sci.

[44] Schneier FR (2009). Dopamine transporters, D2 receptors, and dopamine release in generalized social anxiety disorder. Depress Anxiety.

[45] Cervenka S (2012). Changes in dopamine D2-receptor binding are associated to symptom reduction after psychotherapy in social anxiety disorder. Transl Psychiatry.

[46] de Kwaasteniet BP (2014). Striatal dopamine D2/3 receptor availability in treatment resistant depression. PLoS One.

[47] Larisch R (1997). In vivo evidence for the involvement of dopamine-D2 receptors in striatum and anterior cingulate gyrus in major depression. Neuroimage.

[48] Hesse S (2009). Monoamine transporter availability in Parkinson’s disease patients with or without depression. Eur J Nucl Med Mol Imaging.

[49] Black KJ (2014). Spatial reorganization of putaminal dopamine D2-like receptors in cranial and hand dystonia. PLoS One.

[50] Pickett A (2011). Consistent biochemical data are essential for comparability of botulinum toxin type A products. Drugs R&D.

[51] Dewey SL (1992). GABAergic inhibition of endogenous dopamine release measured in vivo with 11C-raclopride and positron emission tomography. J Neurosci.

[52] Vilkman H (2009). The effects of lorazepam on extrastriatal dopamine D(2/3)-receptors-A double-blind randomized placebo-controlled PET study. Psychiatry Res.

[53] Hou H (2011). Decreased striatal dopamine transporters in codeine-containing cough syrup abusers. Drug Alcohol Depend.

[54] Liu Y (2013). Dopamine transporter availability in heroin-dependent subjects and controls: longitudinal changes during abstinence and the effects of Jitai tablets treatment. Psychopharmacology (Berlin).

[55] Yeh TL (2012). Availability of dopamine and serotonin transporters in opioid-dependent users--a two-isotope SPECT study. Psychopharmacology (Berl).

[56] Zaaijer ER (2015). Effect of extended-release naltrexone on striatal dopamine transporter availability, depression and anhedonia in heroin-dependent patients. Psychopharmacology (Berl).

[57] Tsai HY (2011). Sunshine-exposure variation of human striatal dopamine D(2)/D(3) receptor availability in healthy volunteers. Prog Neuropsychopharmacol Biol Psychiatry.

[58] Yang YK (2008). Decreased dopamine transporter availability in male smokers -- a dual isotope SPECT study. Prog Neuropsychopharmacol Biol Psychiatry.

[59] Thomsen G (2013). No difference in striatal dopamine transporter availability between active smokers, ex-smokers and non-smokers using [123I]FP-CIT (DaTSCAN) and SPECT. EJNMMI Res.

